# Reply: “Comment on: Chocolate Consumption and Risk of Coronary Heart Disease, Stroke, and Diabetes: A Meta-Analysis of Prospective Studies, *Nutrients* 2017, *9*, 688”

**DOI:** 10.3390/nu9080855

**Published:** 2017-08-10

**Authors:** Sheng Yuan, Jinping Lu

**Affiliations:** Department of Geratology, Zhongnan Hospital of Wuhan University, Wuhan University, Wuhan 430071, China; whumedor@163.com

To the editor,

We would like to thank Dr. Hurtado-Torres for his valuable comments on our article entitled “Chocolate Consumption and Risk of Coronary Heart Disease, Stroke, and Diabetes: A Meta-Analysis of Prospective Studies”, which was published in Nutrients in July 2017 [1]. As he pointed out, Buitrago-Lopez et al. [2] also demonstrated the protective effect of chocolate against cardiometabolic disease. However, only one Japanese study published in 2010 [3] was included in their analysis of diabetes, with no quantitative pooling of data. That means Buitrago-Lopez’s description of the benefits of chocolate in preventing diabetes was based on a systematical review, not on a meta-analysis. We therefore stated in the discussion that “our study is the first meta-analysis investigating the protective role of chocolate consumption against diabetes”. 

In addition, Dr. Hurtado-Torres also mentioned an interesting difference regarding the optimum dose of chocolate intake in our and Buitrago-Lopez’s studies. In our highest versus lowest meta-analysis (Figures 2A–4A in Reference [1]) we reached a similar finding as Buitrago-Lopez et al., that is, higher chocolate consumption is associated with lower risk of cardiovascular disease and diabetes. Nevertheless, in our dose-response meta-analysis (Figures 2B–4B in Reference [1]), we found that chocolate consumption in moderation (1–6 servings/week) may be ideal for the prevention of cardiometabolic disease, while no dose-response analyses were performed in Buitrago-Lopez’s study. As categories of chocolate consumption differed between the original studies included, highest versus lowest meta-analysis may complicate the interpretation of the final results. Thus, our study with additional dose-response analyses represents a more accurate evaluation of the relationship between chocolate consumption and risk of cardiometabolic disease. It also should be noted that both our and Buitrago-Lopez’s studies are meta-analyses with inherent limitations; thus, large prospective studies are still required to delimitate the optimal chocolate recommendation. 
